# Response to language barriers with patients from refugee background in general practice in Australia: findings from the OPTIMISE study

**DOI:** 10.1186/s12913-021-06884-5

**Published:** 2021-09-06

**Authors:** Shoko Saito, Mark F Harris, Katrina M Long, Virginia Lewis, Sue Casey, William Hogg, I-Hao Cheng, Jenny Advocat, Geraldine Marsh, Nilakshi Gunatillaka, Grant Russell

**Affiliations:** 1 Centre for Primary Health Care and Equity, Faculty of Medicine, University of New South Wales, New South Wales Kensington, Australia; 2 UNSW Sydney, NSW 2052 Sydney, Australia; 3 Department of General Practice, School of Primary & Allied Health Care, Faculty of Medicine, Nursing and Health Sciences, Monash University, Victoria Melbourne, Australia; 4 Centre for Health Systems Development, Australian Institute for Primary Care and Ageing, La Trobe University, Victoria Melbourne, Australia; 5 Victorian Foundation for Survivors of Torture, Brunswick, Australia; 6 Department of Family Medicine, University of Ottawa, Ottawa, Canada

**Keywords:** Refugees, General Practice, Language barriers, Interpreter use, Practice-wide facilitation

## Abstract

**Background:**

Language is a barrier to many patients from refugee backgrounds accessing and receiving quality primary health care. This paper examines the way general practices address these barriers and how this changed following a practice facilitation intervention.

**Methods:**

The OPTIMISE study was a stepped wedge cluster randomised trial set within 31 general practices in three urban regions in Australia with high refugee settlement. It involved a practice facilitation intervention addressing interpreter engagement as one of four core intervention areas. This paper analysed quantitative and qualitative data from the practices and 55 general practitioners from these, collected at baseline and after 6 months during which only those assigned to the early group received the intervention.

**Results:**

Many practices (71 %) had at least one GP who spoke a language spoken by recent humanitarian entrants. At baseline, 48 % of practices reported using the government funded Translating and Interpreting Service (TIS). The role of reception staff in assessing and recording the language and interpreter needs of patients was well defined. However, they lacked effective systems to share the information with clinicians. After the intervention, the number of practices using the TIS increased. However, family members and friends continued to be used to interpret with GPs reporting patients preferred this approach. The extra time required to arrange and use interpreting services remained a major barrier.

**Conclusions:**

In this study a whole of practice facilitation intervention resulted in improvements in procedures for and engagement of interpreters. However, there were barriers such as the extra time required, and family members continued to be used. Based on these findings, further effort is needed to reduce the administrative burden and GP’s opportunity cost needed to engage interpreters, to provide training for all staff on when and how to work with interpreters and discuss and respond to patient concerns about interpreting services.

**Supplementary Information:**

The online version contains supplementary material available at 10.1186/s12913-021-06884-5.

## Background

Between 2014 and 2019, 15,000 to 20,000 refugees have resettled annually in Australia [1]. A refugee is defined by the 1951 United Nations Convention relating to the state of Refugees as “someone who is unable or unwilling to return to their country of origin owing to a well-founded fear of being persecuted for reasons of race, religion, nationality, membership of a particular social group, or political opinion” [2]. Many arrive with complex physical and mental health and social issues and face challenges to accessing primary health care services [3]. Linguistic and cultural differences between patients from refugee backgrounds and health care providers are major challenges to this population receiving effective primary health care [4–7]. Language barriers may reduce access to quality care from general practice [8], manifesting as missed opportunities for proactive and appropriate care [9, 10]. Language barriers may persist even after resettling for some time in their host country among some people from refugee background with continued need for an interpreter [11]. These barriers may also lead to misdiagnoses or misunderstandings with serious consequences, including the death of a patient or accusations of practitioner negligence [9, 12].

There have been concerted efforts to address language barriers to healthcare access in Australia. These include English language programs [13] and the provision of credentialled interpreter services. The federal government funded the Translating and Interpreting Service (TIS National, hereafter TIS) provides credentialed interpreters via telephone or face-to-face free of charge to medical practices and practitioners for Medicare-funded services [14]. The Royal Australian College of General Practitioners (RACGP) recommends the use of credentialed interpreters in its practice accreditation standards [15, 16].

Yet, the use of TIS was previously reported low at 1 % of Medicare consultations with patients with limited English proficiency in Australia [17], where 33 % of its population was born overseas and 21 % speak language other than English at home. 17 % of the latter group reported not being proficient in English [18]. Instead, family members, friends and relatives or bilingual practice staff continue to be used as interpreters for patients with limited English [9, 19, 20]. This approach affects the quality of health care, posing risks to patient safety due to inaccuracy [19, 21] and raises ethical issues such as confidentiality [21, 22].

Engaging credentialed interpreters has been demonstrated to help improve access and quality of care, uptake of preventive services, compliance to treatment, and satisfaction with care [18, 23, 24] and trust towards the health system [19]. Utilisation of interpreting services may also reduce hospital admissions [19] and lead to long-term cost saving across the health care system [19, 25]. However, to realise these benefits in general practice, changes to clinical and organisational routines are required.

The OPTIMISE study [26] aimed to improve the primary care management of people from refugee backgrounds with outreach facilitation delivered to general practices. It involved a quality improvement process addressing interpreter engagement as one of four core intervention areas. This paper seeks to answer the following questions:

1. How did practices organise to meet the language needs of patients from refugee backgrounds?
2. What changes occurred in practices procedures after the intervention?

## Methods

This study is a mixed-method analysis of quantitative and qualitative data collected at particular time points by the OPTIMISE study. In this section, we describe briefly the methodology of the study as its protocol has been published [26].

### OPTIMISE study

The OPTIMISE study was a stepped wedge cluster randomised controlled trial, set in general practices in three urban regions of high refugee resettlement across two states (Victoria and New South Wales) in Australia, which are culturally diverse and relatively low socio-economic areas [27]. Refugee population in this study includes newly arrived people as well as those who resettled in the country for any length of time.

The study planned to recruit 12 general practices each from 3 study regions, 36 practices in total. Eligibility criteria included practices that (1) provided comprehensive primary health care, (2) used compatible electronic medical record (eMR) software with the study’s data extraction software (PenCAT4™) [28], (3) were in operation at least 12 months, (4) planned to operate at least next 2 years without substantial management changes, and (5) 50 % of Full Time Equivalent (FTE) GPs consented to participate. Recruited practices were then randomly assigned to early and late intervention groups, with stratification by practice size (FTE GPs equal to 5 or below, or above 5) and region.

The OPTIMISE intervention was delivered by outreach facilitators sourced from regional refugee focused health services. As there was a change of staff in one of the study regions, in total 4 facilitators were engaged in the intervention, which included 1 GP and 3 nurses with refugee health experience. Facilitators were asked to make three visits and 3 intervening telephone calls over 3 to 9 months. The intervention used a quality improvement process designed to improve practice conduct of comprehensive health assessments, identification of refugee status, engaging interpreters, and referrals to required health and social welfare services. All practices focused on the same proposed areas by the study. Each facilitator assisted the practice team to follow the quality improvement cycle using action plans as a tool, copies of relevant guidelines and a summary sheet of key resources for refugee health care. In relation to “interpreter engagement”, we prepared a guide on how to record language and interpreter needs in patients’ eMR as commonly used clinical software lacked specific fields to record such information. (See Appendix 1 for more details of the intervention).

The study collected from practice and clinicians using various survey tools information relevant to the intervention such as practice size, languages spoken by staff, practice procedures, performance, and perceived barriers. Further data were collected from patients’ eMR using the data extraction tool PENCS CAT4™. Data on interpreter engagement in consultations was sought from TIS. Qualitative data were collected as free comments in surveys as well as through interviews of participants. These data were collected over four time points (Refer to Table 1).

**Table 1:** Quantitative data used in this study in relation to OPTIMISE data collection points

	T0	T1	T2	T3
Early Group	Baseline	Post intervention	6 months Post intervention	-
Late Group	Baseline	Pre-intervention	Post intervention	6 months Post intervention

Note: Data in shaded time points were included in this study

### Data included in this study

This study utilised a subset of both quantitative and qualitative data related to language preference identification and its recording, and interpreter engagement from practices’ and clinicians’ surveys. Quantitative data for this study derive from T0 and T1 (Table 1). Between these time points, only the early group received the intervention rendering the late group as control. Post intervention qualitative data of both groups (T1 and T2) were used to explore their engagement of interpreters, attitudes, barriers to engaging interpreters, and better understand the reasons for trends in the quantitative data.

In total, this study included quantitative and qualitative data from 31 practices which were randomised into the early and late groups, as well as data of 55 GPs from these practices who responded to clinician surveys at both T0 and T1 (Table 2).

**Table 2:** Composition of Early and Late groups included in this study

	Early/ Intervention Group	Late/ Control Group	Total
Practices	17	14	31
GPs	36	19	55

### Data analysis

Descriptive analysis and nonparametric tests of quantitative data were carried out using IBM SPSS Statistics (ver.26) [29]. For practice level data, McNemar and Wilcoxon Signed Rank tests were used to examine pre-post intervention changes within groups, while difference in differences (DiD) analysis with linear regression was used for examining changes between groups. For practitioner level data, analyses were adjusted for possible clustering effects at the practice level using Generalized Estimating Equations (GEE). The significance level for the statistical tests was set at 0.05.

Language concordance was assessed by comparing languages spoken by each GP with those of their patients from refugee backgrounds reported in the clinician survey. If the GP spoke any of the prevalent patients’ languages, then it was assessed as either fully or partially language concordant. For example, if a GP spoke Dari and Dari was one of the common languages spoken by their refugee background patients, then this GP was partially language concordant. They would be considered fully language concordant if all their refugee background patients spoke Dari. Language data were self-reported by GPs.

The qualitative data was coded initially using Nvivo (ver.12) [30] based on theoretical concepts from Stange and Glasgow’s Context Tool [31] and May’s Normalisation Process Theory [32] by qualitative researchers in the study team (KL, SD, SV, SJ). Text coded as relating to language or interpreter engagement were then thematically recoded to identify patterns of use, barriers and enablers by two members of this study team (SS and MH).

## Results

The OPTIMISE study team approached 88 practices of which 35 practices met eligibility criteria. These were assigned randomly to the early group (18 practices) or to the late group (17 practices). Four of them withdrew prior to the completion of the study, leaving 31 practices (17 in the early group and 14 in the late group). The baseline data are presented for all practices as there was no statistical differences in the early and late groups in the reported items at this point in the study.

### General and linguistic characteristics of participating practices and GPs

Of 31 practices, 25 practices had less than 4 full time equivalent (FTE) GPs and 6 had four or more FTE GPs, with a mean of 2.6 FTE GPs. The mean percentage of patients from refugee backgrounds per practice was 6.2 % (range: 0.04–86.4 %) in the last 5 years with a median of 156 patients per practice. The number of patients from refugee backgrounds who visited the practices in the 12 months period prior to the intervention varied widely ranging from 7 to 1,781 per practice. Most GP participants perceived that a large proportion of their patients from refugee backgrounds were not fluent in English.

All practices had at least one bilingual GP or staff and 22 practices (71 %) had at least one GP who spoke the language spoken by recently arrived people from refugee backgrounds to Australia [32]. Many of the practice personnel were bilingual or multilingual, GPs being the highest proportion followed by reception staff, allied health practitioners and nurses. However, not all those bi or multilingual personnel necessarily spoke the languages spoken by recently arrived refugees (Table 3).

**Table 3:** Practice personnel speaking languages other than English (LOTE) and languages spoken by recently arrived refugees^1^

		Speak LOTE^2^			Speak Refugee language^1,3^		
Practice personnel	N	Yes (n)	%	95 % CI	Yes (n)	%	95 % CI
GPs	143	122	85.3	69.3–78.4	62	43.4	35.1–40.0
Nurses	68	34	50.0	44.8–90.7	8	11.8	51.9–22.1
Reception/Admin	142	87	61.3	37.8–64.2	45	31.7	5.2–40.2
Allied Health	108	59	54.6	61.0–62.4	33	30.6	21.9–27.9
All	461	302	65.6	52.7–69.8	148	32.8	24.1–36.3

Data Source: Practice Description Survey (PDS)

^1^Refugee languages are based on patients’ country of origin or ethnicity of the source countries of recent humanitarian entrants to Australia as reported by GPs (Department of Immigration and Border Protection, 2013 to 2017 [33]

^2^Chi-square test of “Speak LOTE” by practice staff *p* = 0.000

^3^Chi-square tests of “Speaks Refugee language” by practice staff *p* = 0.000

GPs in this study were from 19 different countries, with 89 % born outside Australia. Of these, 75 % arrived in Australia after the year 2000. Table 4 indicates most common languages spoken by GPs and by their patients from refugee backgrounds that were reported by GPs. When comparing the languages spoken by GPs and their patients with refugee background, available data suggested a moderate level of language concordance between GPs and this patient group.

**Table 4:** Top five languages spoken by patients (as reported by GPs) and by GPs

Ranking	Spoken by GPs	Reported by GPs as spoken by their refugee patients
1	Arabic (18)	Arabic (32)
2	Hindi (9)	Persian/Farsi (15)
3	Urdu (6), Bengali (6)	Assyrian (9)
4	Persian (5)	Dari (8), Urdu (8)
5	Dari (3), Cantonese (3)	Bosnian (7)

Data Source: Clinician Survey at T0, Numbers in brackets are frequencies of responses

### Practice organisation and performance at the baseline

#### Practice set-up and training for the use of TIS

All except one practice in the early group were registered with TIS, with most participating GPs having TIS registration. Fifty percent of all practices displayed the poster indicating the availability of the TIS service and the number to call in the waiting room. Ninety-seven percent of practices had a speaker phone in consultation rooms and 19 % had TIS phone numbers placed on phones or nearby. While 9 % of reception staff reported being offered training on how to work with interpreters by the practice, none of the GPs in either group had been offered such training, despite being the main users of interpreting services.

#### Assessment and recording of interpreter needs

In most practices, it was the responsibility of reception and administration staff to establish the need for, and to book an interpreter. (see Table A-1 in the appendix 2). The qualitative data suggested that language preferences and the need for an interpreter were most often sought by reception staff when a patient have difficulties in communicating in English or when completing a new patient registration form. This was also sometimes identified during GP or nurse consultations. The staff also used a variety of methods to establish the need for an interpreter, such as patients’ statement on the patient registration form, being told by family or friends accompanying the patient, referral notes from refugee health or settlement services, patient gesturing to a TIS poster, use of language cards, or when patients struggled to complete the registration form.

Despite having practices set up to provide TIS and the reception staff or GPs having established the language preference and interpreter needs of patients, few practices had a routine system or procedure to share such information across all staff.

*“The Dr may include need for interpreter in progress notes, not a procedure followed by all Drs or staff. Unsure where it is recorded in the clinical notes …”* [Comment in Refugee Health Survey (RHS) at T0]

Having no specific field to record the information in eMR software was reported as a major barrier to recording need in this situation.

#### Self-reported use of TIS

Seventy-seven perecent of GPs reported having no difficulty in providing care to patients who spoke a different language to their own, and 69 % reported that using a credentialed interpreter did not interfere with the delivery of health care. GPs perceived a large proportion of their patients from refugee backgrounds had limited English proficiency yet reported using TIS with only 21 % of those patients. Language concordant GPs tended to use TIS less frequently (9 %) compared to non-language concordant GPs (27 %) (NS). At the practice level, 23 % of practices perceived no need to use TIS because the GPs shared the same language with the patient group. 29 % of practices reported not using it.

*“Hasn’t been discussed at the practice because Drs speak Arabic and don’t need to use interpreters. The refugees that come to this practice speak Arabic. GPs don’t believe they need to use an interpreter.”* [Practice Manager (PM), Interview at T1]

When telephone based and face-to face modes of interpreter services were combined, 48 % of practices used TIS prior to the intervention, while all but one of the practices were registered with the service. Qualitative interviews suggested that being registered with TIS alone did not necessarily facilitate the use of TIS when required because of the availability of bilingual staff or family members/friends accompanying patients.

*“Although GP has been registered with TIS, it has never been used so far. Patients always come with someone who speaks English”* [Comment in RHS at T0].

Some practices reported that their GPs and other staff were proficient in a range of languages and language was a criterion in recruiting new GPs or other staff.

“*We need bilingual GPs when you’ve got a lot of patients and some of them don’t speak English at all. It is always good idea to have staff here that can speak at least two different languages.”* [PM, Interview at T2].

#### Alternative means to engaging credentialed interpreters

In the baseline clinician survey, besides credentialed interpreters, 50 % of GPs reported using bilingual staff, online translation apps such as “Google translate” and friends or family of patients to communicate with patients from refugee backgrounds with limited English proficiency. One practice indicated that children were used to interpret.

#### Barriers to engaging interpreters reported by GPs

**Fig. 1:**
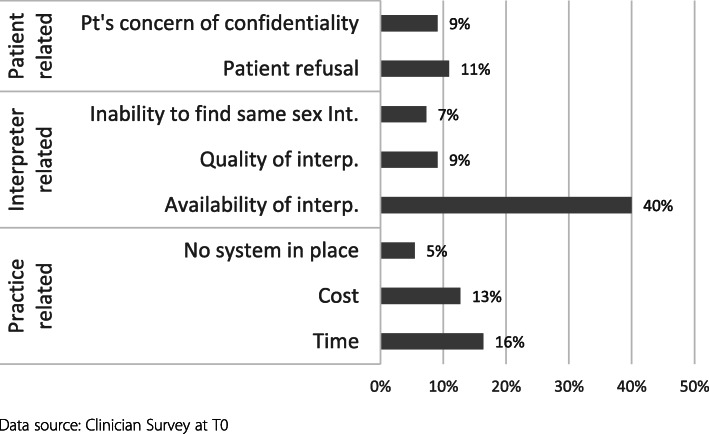
Barriers reported by GPs (*n* = 55, Multiple responses, Baseline, Groups combined)

Fifty-three of 55 GPs reported barriers to using interpreting services. These included barriers within the practice, interpreter services and perceived patient related barriers (Fig. 1). The availability of interpreters was reported as the major barrier by both groups (44 % of the early group and 53 % of the late group). This was followed by time and cost.

In qualitative interviews and text responses, GPs cited patients’ reluctance due to concerns about confidentiality or the desire to use family members as a barrier.

*“Even if I want to do that (use interpreter), the patients bring in family member. So, that is something to do with culture, you know….They do like if it’s a sister or a brother they can tell them what’s the problem.”* [GP, Interview at T2].

GPs mentioned the extra time involved in consultations and lack of specific funding to enable this.

*“Takes too long to do a consultation with an interpreter present. Quality and accuracy of interpretation does not meet my expectations. Sometimes the patients talk for 5 minutes, and the interpreter sums it up in a few short sentences.”* [GP, interview at T2].

One practice mentioned patients’ lack of punctuality as a potential issue.

*“The practice did not realise they could pre-book interpreters through TIS. They say that this would not be useful anyway as patients do not turn-up at the right time or even on the right day.*” [Research officer’s comment in RHS, T0].

In contrast with the quantitative data, the availability of interpreters did not feature prominently in interviews or free text responses except for noting the difficulties in finding interpreters of some dialects, for certain languages or during after-hours.

### Changes observed after the intervention

Here, the early group received intervention and the late group is the control.

#### Changes to practice set-up and training for the use of TIS

There was an upward trend in the proportion of practices in the early (intervention) group displaying information about the availability of interpreters via TIS (56 to 88 %); having a speaker phone in consultation rooms (88–100 %); and having the TIS sticker on the phone or nearby (12–50 %). A practice manager reported that making information available about TIS for the patients led to patient-driven increase of interpreter service utilisation.

*“There is a poster now on the wall in the waiting room to increase patients’ awareness of this service. This has led to the patients driving the interpreter use rather than GPs.”* [PM, interview at T1].

The late (Control) group remained unchanged in all these aspects (at 43 %, 100 and 28 % respectively). One practice in the early group reported a nurse trained the admin staff how to record patient’s language and interpreter need into eMR using OPTIMISE resources, but no practices initiated training on how to work with interpreters or discussing use of interpreting services with patients.

#### Changes to assessment and recording of interpreter need

**Fig. 2:**
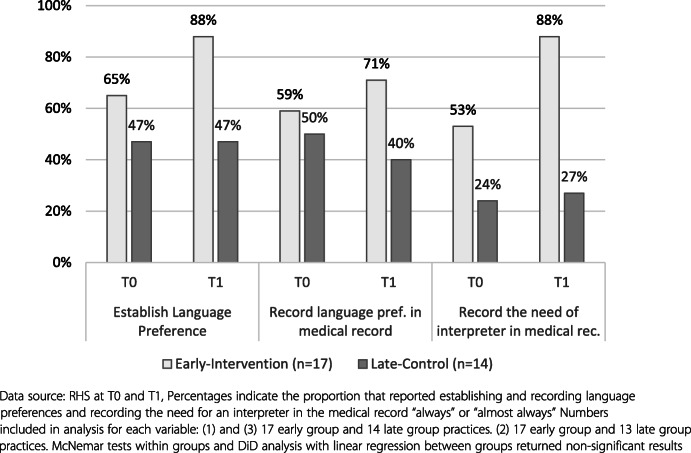
Performance of language related tasks by group

The early group demonstrated a trend of improvement in collecting and recording language needs, while the late group remained at similar levels (Fig. 2).

Qualitative data suggested that the trend for improvement could be attributed to changes to the new patient registration form, promoted by the intervention.

*“Because we changed the patient registration form, so they are much more onto whether an interpreter is needed or that sort of screening process that happens initially when they’re first coming in…which they wouldn’t have taken much notice*.” [PM, Interview at T1].

After the intervention, practice staff were more confident in asking about and recording interpreter needs and scheduling interpreters following the intervention. This made reception staff more relaxed about asking this information of patients.

However, as noted earlier, no specific recording field for language and the need for interpreter in the eMR software was a barrier to making changes for some practices. Because of this one practice reported not having changed the registration form. A change in the software to accommodate such information would facilitate improving the practice procedure as reflected in the following quote.

*“I will recommend to have a box to tick in Medical Director (software) . it will become easier for doctors to provide the care according to their needs”* (PM, Interview at T1).

Some were surprised by how easy it was to arrange an interpreter – even a face-to-face interpreter - and the intervention raised awareness on the benefits of engaging interpreters.

*“I think it raised awareness about involving interpreters early not just when it’s like last ditch or that kind of thing. And that is always going to result in a higher quality of care when the patient actually understands what’s happening in the consultation, rather than just bare bones understanding*” [Nurse, Interview at T2].

Confidence in arranging interpreter services increased significantly among GPs in the early group but not in the late group (see Table A-2 in the Appendix 2). However, this change was not significant between groups. As most bookings with TIS are arranged by reception staff, this may reflect increased knowledge about TIS among GPs.

#### Changes in self-reported engagement of credentialed interpreters

**Fig. 3:**
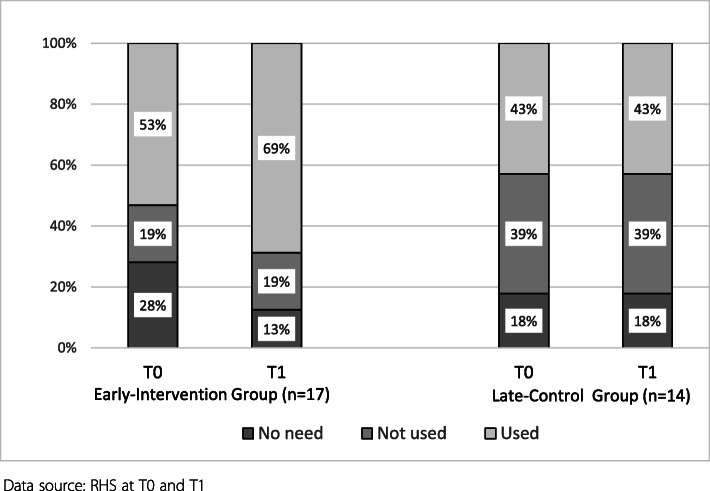
Changes in practice-reported usage of Interpreters (TIS-both modes combined)

The number of practices in the early group using TIS increased from 9 to 12 while the late group remained the same (Fig. 3) (NS). This trend was consistent with TIS service utilisation data, which indicated the total number of interpreters engaged d in the early group increased from 101 to 174 during the intervention period, while that of the late group changed very little from 42 to 45 during the same period.

#### Continued use of alternatives to engaging credentialed interpreter**s**

After the intervention, 37 % of practices reported that other bi-or multi-lingual staff were used to interpret. A further 28 % of practices reported using family members or friends to interpret. “Patients’ preference” or “Patients’ request” were common reasons for using this option. In qualitative interviews, some GPs reported using family members especially where patients requested it. However, they talked about doing this only for consultations about physical health but not for mental illness.

*“I asked (about using interpreter to patients) but they say no because we don’t know who is on the phone, especially for mental health… It helps when patient is not really worried about confidentiality, like a headache.”* [GP, Interview at T1].

One GP was more cautious – asking patients to give specific consent to use a family member.

“*Because I’m from the same culture, I understand they feel comfortable if I let them (family members) interpret….But I make sure that I write verbal consent from patients because it’s about confidentiality, (so that) they don’t come back and say “I didn’t want my brother in consultation*”.” (GP, Interview T1).

#### Changes in barriers to the use of TIS reported by GPs

The availability of interpreters remained the most frequently cited barrier by GPs in both early and late groups. The proportion of GPs describing cost and availability of interpreters as barriers decreased significantly in the early group (17–0 %, *p* = 0.021 and 44–28 %, *P* = 0.043 respectively by GEE) compared to the late group. In contrast, the number of GPs reporting “no barriers” in the early group increased significantly (3–29 %, *p* = 0.036 by GEE) (see the table A-3 for details in the Appendix 2).

However, participants were concerned about the time involved in arranging interpreters and, if arranged in advance, the wasted effort when patients did not attend.

*“(for) a long appointment, admin staff had to book two interpreter timeslots. So, appointment was a biggest thing we had to coordinate”.* (PM, Interview T1)

*“(We) Started to book interpreters. But it brought its own problems when people didn’t turn up.”* (GP Interview, T1).

## Discussion

General practices employed several means to respond to language barriers between refugee background patients and health care providers. We found a high proportion of bilingual GPs and other staff across our study practices and moderate levels of apparent language concordance between GPs and their refugee background patients. This may be the result of patients from refugee backgrounds seeking GPs who speak the same language [9], and the practices responding to such demand by hiring more bilingual GPs and other staff. While the availability of language concordant GPs and staff, who are proficient in the language, is beneficial to patients and practices, this has potential implications for the general practice workforce and regional and rural areas where an increasing proportion of refugees are being resettled in Australia [34].

Self-reported practice data indicated that half of the practices did not use or perceive any need for interpreter services prior to the intervention. An obvious factor influencing this behaviour is the wide availability of bilingual GPs in practices, which is consistent with other studies [9, 16, 35]. Half of practices reported using alternative means of communication such as family members and friends, other bilingual staff, or online translation. This is contrary to RACGP accreditation standards which state that practices should “use an interpreter with patients who do not speak the primary language of our practice team” (C1.4 A) [15].

Patient’s preference for having family members to interpret was another factor GPs and practice staff identified as contributing to a low rate of credentialed interpreter engagement. In addition to lack of familiarity with services, for patients from refugee backgrounds, reluctance to engage interpreters may be due to a concern at having someone they do not know or trust in the consultation in the context of a history of political, religious or ethnic persecution in their own country [9, 36, 37]. Mistrust of authority figures due to their experiences [8, 36] and/or fear of lack of confidentiality within their community [37] may discourage their use of interpreting services. Patients often bring family members with them for interpreting and support. This may lead to a misconception that using family members for interpreting is the patients’ preference and it is their responsibility to bring them [16, 38].

Time pressure and related opportunity costs to GPs and practices is another driver for not using interpreters. Although TIS itself is free of charge, it requires additional waiting and consultation time, in addition to administrative time for booking the service [19]. The difficulties associated with finding an interpreter and poor quality based on perception or past negative experience also discouraged the use of interpreter services [19, 39]. There is a strong case for this additional time requirement to be recognised in the funding of all consultations through Australia’s Medical Insurance Scheme (Medicare) when interpreting services are engaged.

Engaging a credentialed interpreter is a crucial patient safety issue and affects the quality of care they receive. Although family members may have a role to play [40], they are not an acceptable alternative to credentialed interpreters as they are likely to lack understanding of medical conditions, terminology, and medications with potential risk to safety [8, 41–43]. Family members’ presence may interfere with sharing of sensitive information such as sexual health [8, 9, 22, 44]. Bilingual staff may not be proficient in translating medical terminology or they may lack an understanding of the interpreter role in providing accurate and impartial messages [19, 45]. Although online translation may play a role as technology advances, its use is not currently recommended due to concern over accuracy [46, 47] and security in Australia [47]. At the same time, the professional status and employment conditions of interpreters need to be looked into to ensure the availability of qualified interpreters [48].

It is important that formal procedures are in place to ensure patients from refugee backgrounds are routinely offered free credentialed interpreter services for all consultations, the confidentiality of interpreter services explained, and any other concerns addressed [16, 21, 36]. To improve the engagement of credentialed interpreters, a practice-wide system is required to coordinate the different roles of practice staff in relation to providing quality care. The decision-making process to engage an interpreter needs to be clear to avoid relegation to “nobody’s business” [35]. Receptionists are the first point of contact with patients and have a key role in identifying the language and needs of the patient for an interpreter. The patient’s requirement for an interpreter needs to be shared across all staff by, for example, agreeing on a specific and visible place to record such a requirement in the patient’s electronic medical record (eMR). However, the most frequently used eMR in Australian general practice do not provide a specific field for recording interpreter requirements or refugee status. This could be addressed by software manufacturers providing these fields in support of accreditation standards.

### Impact of the OPTIMISE intervention on interpreter engagement and implications

The OPTIMISE intervention encouraged the identification of language and interpreter needs and recording of these data by receptionists. Increased awareness, improved procedures and the reduction of perceived barriers by GPs in the intervention group contributed to this trend. The number of practices self-reporting the engagement of credentialed interpreters improved in the early (intervention) group, although this was not statistically significant because of the small number of practices involved in the study. This suggests the need for larger scale implementation research in general practice if such measures are implemented at scale by primary health care networks and if complementary system level changes are introduced.

The absolute level of the uptake of TIS remained very low as also indicated by other studies [8, 9, 17]. This suggests the need for an approach which is tailored to the needs of each practice (including the language proficiency of staff). The OPTIMISE intervention provides a starting point for how support can be provided as it engaged a whole of practice approach and built on existing Primary Health Network (Australian primary health care organisations) refugee outreach programs to support practice staff. Based on the observations of facilitators involved in implementing the OPTIMISE intervention and an earlier paper by Phillips [16], we recommend 10 actions to general practices for building a practice-wide system to promote the interpreter engagement and to be included in refugee practice support programs.

***Ten actions for building a practice-wide system to promote the engagement of credentialed interpreters at the general practice setting.***

1. Practice registration with TIS (all GPs to have a code. These codes to be shared with receptionists and nurses, advising them to access free interpreting when organizing appointments and coordinating care utilizing the codes. (TIS National Registration -https://tisoline.tisnational.gov.au/RegisterAgency)
2. Poster in multiple languages encouraging patients to indicate their preferred language, with information that TIS is free and interpreters respect confidentiality.
3. Have a system to check language preference of patients at reception and record in electronic Medical Record (eMR).
4. Having a speaker phone in each clinical room (including nurses and allied health) with the TIS phone number and GP/Practice codes stuck on it.
5. Decision making process within the practice to be agreed upon by all staff regarding who should initiate and book the interpreter service.
6. Training all staff on: benefits of using credentialed interprets; risks of not doing so; how to arrange an interpreter; and how to work with an interpreter in consultations.
7. Training all staff on how to encourage patients/families to engage credentialed interpreters.
8. Auditing languages spoken by patients in the practice and receiving information from local the refugee health service on languages spoken by recently arrived refugees. Using this to plan how to improve language appropriate care by the practice.
9. Preparing a list of languages spoken by referral practitioners outside of the practice (medical specialists, allied health).
10. Providing referral navigation information in multiple languages (e.g.; how to book and get to referral service etc.) either in writing or using a health navigator.

Various models of outreach from refugee health services into general practice may facilitate achieving this engagement [49]. However, to make this change sustainable, broader systematic changes are needed including more rigorous implementation of practices’ readiness to engage credentialed interpreters in accordance with the accreditation standards for general practices and the review of the Medicare rebate for interpreted consultations as it does not adequately reimburse the practice for the increased time involved in consultations and administration [19]. As noted above there also need to be changes to the eMR in use in general practice. Ideally these interventions should be codesigned with interpreters and CALD community organisations, themselves as well as GPs and other general practice staff.

### Limitations of the study

There were limitations to the study. The intervention was carried out and evaluated largely at the level of the practice. This meant that our analyses were underpowered to detect even moderate statistically significant changes.

The data such as confidence, barriers, practice procedures relied on retrospective self-report by GPs and practice staff which may result in reporting bias. Limitations also include our inability to accurately determine language needs of the patients and therefore to determine the concordance of GP languages due to limited recording of data in patient’s eMR and self-reporting nature of the clinicians’ data.

## Conclusions

General practices in our study responded to the language barriers between patients from refugee backgrounds and clinicians by hiring bilingual GPs and staff, using TIS as well as bilingual staff, family members and online translation despite these alternatives being against current best practice. Barriers to use of TIS reported by GPs included the perceived poor availability and quality of interpreters as well as patients’ preference and concern about confidentiality. While TIS provides free of charge interpreter service for GPs for Medicare funded consultations in Australia, the opportunity cost for GPs in terms of the available time was identified as a significant barrier and needs to be addressed at a system level.

The practice-wide intervention facilitated practices to improve the identification and recording of language preferences and interpreter needs as well as engagement of interpreting services. Strong engagement of both reception staff and clinicians has the potential to improve this further. Training on how to work with interpreters and how to discuss the engagement of interpreters with patients and their families are also required. However further research at scale is needed, focussing on how the intervention should be tailored to the needs of each practice and the system change that is required to support this.

## Supplementary Information

**Additional file 1: Appendix 1.** Additional information on the OPTIMISE intervention. **Appendix 2: Table A-1.** Responsibility of establishing the need for and booking an interpreter by group (Multiple responses, Baseline). **Table A-2.** Confidence of GPs arranging an interpreter. **Table A-3.** Perceived barriers related to the engagement of credentialed interpreters before and after intervention reported by GPs (Multiple responses)

## Data Availability

De-identified versions of the datasets generated and analysed for this study, the statistical analysis plan and relevant statistical code will be available on request (see request procedure in the Data Management Plan at https://bridges.monash.edu/articles/data_management_plan/OPTIMISE_Data_Management_Plan/13359068). Availability will begin 6 months after and end 36 months following article publication. The original data are not publicly available due to them containing possibly re-identifiable participant information.

## Abbreviations

GP: General Practitioners
TIS: Translating and Interpreting Service
RACGP: Royal Australian College of General Practitioners
eMR: Electronic Medical Record
LOTE: Languages Other Than English
