# Supplementary material for: Response to language barriers with patients from refugee background in general practice in Australia: findings from the OPTIMISE study
Source: BMC Health Serv Res. 2021 Sep 6;21:921. doi: 10.1186/s12913-021-06884-5 (PMC8419978; doi:10.1186/s12913-021-06884-5)
Supplement: Supplementary file 1 — Additional file 1: Appendix 1. Additional information on the OPTIMISE intervention. Appendix 2: Table A-1. Responsibility of establishing the need for and booking an interpreter by group (Multiple responses, Baseline). Table A-2. Confidence of GPs arranging an interpreter. Table A-3. Perceived barriers related to the engagement of credentialed interpreters before and after intervention reported by GPs (Multiple responses) [file 12913_2021_6884_MOESM1_ESM.docx]

**Appendix 1: Additional information on the OPTIMISE intervention**

The OPTIMISE project aimed to build capacity of general practices to respond to the needs of people of refugee background through a practice facilitation intervention.

The intervention was delivered between November 2017 and March 2019 in 31 general practices; 11 practices each in South West Sydney and North West Melbourne and nine in South East Melbourne.

Practice facilitators used a quality improvement approach and worked with practices over an approximately six-month period supporting them to implement an action plan (following a pre-designed template) focused on the four areas targeted by OPTIMISE; to improve refugee identification, interpreter use, comprehensive health assessment and referrals. There were four facilitators involved due to a staffing change, three were nurses with refugee health experience of which one worked for a local refugee health agency as a project officer, and one was a GP with a special interest in refugee health care. Prior to the commencement of the intervention, the facilitators attended a one-day training session about practice facilitation.

The pre-populated practice action plans were modified by the facilitators after discussing and identifying issues and possible solutions with practices. Each practice was asked to establish an OPTIMISE staff team responsible for the implementation of the intervention. It was recommended that the team include at least one GP, the practice manager and (if applicable) one practice nurse. Practices identified a staff member to be the key contact for the facilitator, who would also be responsible for ensuring engagement of practice staff in implementing the action plan activities. In accordance with the protocol, all but one practice received at least three, 60-90 min, in-person contacts with a facilitator. Not all practices received the planned telephone contacts following each visit, despite them being scheduled. In many instances phone calls were replaced by ad hoc or planned practice visits.

Practices were provided with additional resources relating to the four core priority areas, including a soft and hard copy of a “General Practice Resource Book” and a two-page electronic ‘summary sheet’ of key regional resources for refugee health care, as well as instructions on how to record refugee status, preferred language and interpreter needs on the patients’ electronic medical record.

Facilitators documented their visits in facilitator diaries and tracked the implementation of action plans at the practices. They participated in regular on-line meetings during the project with other facilitators and research officers from the other regions to share the progress and to discuss strategies to address emerging issues. Each region appointed a research officer to recruit practices, collect study data, provide support and guidance to the facilitator and deal with issues raised by practices throughout the intervention.

Further details about implementation of the intervention will be provided in a future publication.

**Appendix 2:**

Table A-1: Responsibility of establishing the need for and booking an interpreter by group (Multiple responses, Baseline)

|  | Early Group (n=17) | | Late Group (n=14) | | Total (31) |
| --- | --- | --- | --- | --- | --- |
|  | n (%) | 95% CI (%) | n (%) | 95% CI (%) | n (%) |
| ***Establishing the need of an interpreter*** | | | | | |
| Not done | 0 ( 0.0) | 0.0 – 19.5 | 2 (14.3) | 1.8 –42.8 | 2 ( 6.5) |
| Doctors | 11 (64.8) | 38.3 – 85.5 | 6 (42.9) | 17.7 – 71.1 | 17 (54.8) |
| Nurse | 9 (52.9) | 27.8 – 77.0 | 4 (28.6) | 8.4 - 58.1 | 13 (41.9) |
| Practice manager | 5 (29.4) | 10.3 – 56.0 | 2 (14.3) | 1.8 – 42.8 | 7 (22.6) |
| Reception/Admin | 13 (76.5) | 50.1 – 93. | 7 (50.0) | 23.0 – 77.0 | 20 (64.5) |
| Others | 1 ( 5.6) | 1.0 – 28.7 | 0 ( 0.0) | 0.0 – 23.2 | 1 ( 3.2) |
| ***Booking an interprete****r* | | | | | |
| Not done | 1 (5.9) | 1.0 – 28.7 | 2 (14.3) | 1.8 – 42.8 | 3 (9.7) |
| Doctors | 4 (23.5) | 6.8 – 49.9 | 4 (28.6) | 8.4 – 58.1 | 8 (24.8) |
| Nurse | 4 (23.5) | 6.8 – 49.9 | 2 (14.3) | 1.8 – 42.8 | 6 (19.4) |
| Practice manager | 4 (23.5) | 6.8 – 49.9 | 1 (7.1) | 0.2- 33.9 | 5 (16.1) |
| Reception/Admin | 14 (82.2) | 56.6 – 96.2 | 9 (64.3) | 35.1- 87.2 | 23 (72.2) |
| Others | 0 (0.0) | 0.0 – 19.5 | 0 (0.0) | 0.0 – 23.2 | 0 (0.0) |

Data source: PDS, 31 practices. No statistical difference between groups.

Table A-2: Confidence of GPs arranging an interpreter

|  | **Early (Intervention) Group** | | | | **Late (Control) Group** | | | |
| --- | --- | --- | --- | --- | --- | --- | --- | --- |
|  | Before | | After | | Before | | After | |
|  | n (%) | 95% CI (%) | n (%) | 95% CI (%) | n (%) | 95% CI | n (%) | 95% CI (%) |
| Not confident | 9 (32.2) | 18.0 - 51.8 | 3 (10.7) | 3.6 - 29.8 | 8 (47.1) | 23.0 - 72.2 | 5 (29.4) | 10.3 - 56.0 |
| Confident | 19(67.9) | 48.2 - 82.0 | 25(89.3) | 70.2 - 96.4 | 9 (52.9) | 27.8 - 77.0 | 12 (70.6) | 44.0 - 89.7 |
| Total | 28 |  | 28 |  | 17 |  | 17 |  |

Data Source: PS. Although values of 95% CI overlaps partially in “moderately to very confident” in the early group, McNemar test for the same group returned p=0.016. GEE returned non-significant result.

Table A-3: Perceived barriers related to the engagement of credentialed interpreters before and after intervention reported by GPs (Multiple responses)^1),2)^

|  | **Before intervention** | | | **After intervention** | | |
| --- | --- | --- | --- | --- | --- | --- |
|  | n (%) | 95% CI (%) | | n (%) | 95% CI (%) | |
| ***Early Group (n=36)*** |  |  | |  |  | |
| Barriers  Cost  Time^1)^  No system in place  Availability of interpreter  Inability to get same sex interpreter  Quality of interpreters  Patient refusal  Patient concern re confidentiality  No barriers | 6 (16.7)  6 (16.7)  2 (5.6)  16 (44.4)  1 (2.8)  3 (8.3)  5 (13.9)  2 (5.6)  1 (2.8) | 6.4 – 32.8  6.4- 32.8  0.7- 18.7  27.9- 61.9  0.1- 14.5  1.8- 22.5  4.7- 29.5  0.7 - 18.7  0.1 – 14.5 | | 0 (0.0)  4 (11.1)  1 (2.8)  10 (27.8)  1 (2.8)  2 (5.6)  5 (13.9)  5 (13.9)  7 (29.4) | 0.0 – 9.7  0.8- 21.4  0.1 – 14.5  14.0 – 45.2  0.1- 14.5  0.7 – 18.7  4.7 – 29.5  4.7 – 29.5  8.2- 36.0 | |
| ***Late Group (n=19)*** |  |  |  |  |  |  |
| Barriers  Cost  Time^1)^  No system in place  Availability of interpreter  Inability to get same sex interpreter  Quality of interpreters  Patient refusal  Patient concern re confidentiality  No barriers | 1 (5.3)  1 (5.3)  0 (0.0)  6 (31.6)  3 (15.8)  2 (10.5)  1 (5.3)  3 (15.8)  1 (5.3) | 0.1 - 26.0  0.1 - 26.0  0.0 - 17.6  12.6 - 56.6  3.4 - 39.6  1.3 - 33.1  0.1 - 26.0  3.4 - 39.6  0.1 - 26.0 | | 2 (10.5)  0 (0.0)  1 (5.3)  9 (47.4)  5 (26.3)  2 (10.5)  4 (21.1)  4 (21.1)  0 (0.0) | 1.3 - 33.3  0.0 - 17.6  0.1 - 26.0  24.4 - 71.1  9.1 - 51.2  1.3 - 33.1  6.1 - 45.6  6.1 - 45.6  0.0 - 17.6 | |

Data source: Clinician survey. 1) Data on Time was computed from comments, while other items were pre-determined response options.
